# Supplementary material for: Multi-omics analyses reveal that the gut microbiome and its metabolites promote milk fat synthesis in Zhongdian yak cows
Source: PeerJ. 2022 Dec 2;10:e14444. doi: 10.7717/peerj.14444 (PMC9744170; doi:10.7717/peerj.14444)
Supplement: Supplemental Information 13 [file peerj-10-14444-s013.zip › Web_Report/HTML/meta_hmdb_anno_categroy.xls.html]

meta\_hmdb\_anno\_categroy.xls


## meta\_hmdb\_anno\_categroy.xls

| SuperClass | HMDB\_taxonomy | Metabolites\_nums | Metabolites |
| --- | --- | --- | --- |
| Alkaloids and derivatives | Aporphines | 3 | pos\_1566 pos\_1650 pos\_1447 |
| Alkaloids and derivatives | Morphinans | 1 | pos\_1447 |
| Alkaloids and derivatives | Phthalide isoquinolines | 2 | pos\_1566 pos\_1447 |
| Benzenoids | Benzene and substituted derivatives | 18 | pos\_1874 pos\_2710 pos\_1427 pos\_1138 pos\_2788 pos\_363 pos\_2827 pos\_41 pos\_492 pos\_368 pos\_22 pos\_2536 pos\_1750 pos\_2385 pos\_2048 pos\_1622 pos\_112 pos\_159 |
| Benzenoids | Phenol ethers | 27 | pos\_2710 pos\_22 pos\_2314 pos\_2826 pos\_2827 pos\_1254 pos\_368 pos\_982 pos\_2536 pos\_492 pos\_2048 pos\_1427 pos\_1138 pos\_786 pos\_1398 pos\_2385 pos\_1874 pos\_36 pos\_1392 pos\_2788 pos\_363 pos\_2850 pos\_41 pos\_1750 pos\_1622 pos\_112 pos\_159 |
| Benzenoids | Phenols | 25 | pos\_2710 pos\_22 pos\_2314 pos\_2826 pos\_2827 pos\_1254 pos\_368 pos\_2536 pos\_492 pos\_2048 pos\_1427 pos\_1138 pos\_786 pos\_2385 pos\_1874 pos\_36 pos\_1392 pos\_2788 pos\_363 pos\_2850 pos\_41 pos\_1750 pos\_1622 pos\_112 pos\_159 |
| Homogeneous non-metal compounds | Non-metal oxoanionic compounds | 1 | pos\_2612 |
| Hydrocarbons | Unsaturated hydrocarbons | 2 | pos\_2474 pos\_296 |
| Lignans, neolignans and related compounds | Flavonolignans | 1 | pos\_1388 |
| Lignans, neolignans and related compounds | Lignan glycosides | 2 | pos\_2568 pos\_1388 |
| Lipids and lipid-like molecules | Fatty Acyls | 119 | pos\_1492 pos\_1965 pos\_1252 pos\_1510 pos\_2382 pos\_1711 pos\_1005 pos\_827 pos\_560 pos\_250 pos\_627 pos\_2292 pos\_1131 pos\_664 pos\_2277 pos\_2744 pos\_800 pos\_2560 pos\_1998 pos\_1493 pos\_1201 pos\_1570 pos\_2760 pos\_577 pos\_2356 pos\_1717 pos\_1442 pos\_2759 pos\_37 pos\_2480 pos\_2484 pos\_809 pos\_779 pos\_2800 pos\_2578 pos\_2169 pos\_2819 pos\_522 pos\_2679 pos\_1041 pos\_1271 pos\_796 pos\_1426 pos\_1180 pos\_2351 pos\_2844 pos\_2725 pos\_1904 pos\_604 pos\_2419 pos\_2732 pos\_2369 pos\_1421 pos\_1790 pos\_722 pos\_1861 pos\_633 pos\_1366 pos\_932 pos\_2166 pos\_554 pos\_2000 pos\_2557 pos\_1026 pos\_2236 pos\_52 pos\_1097 pos\_2068 pos\_1147 pos\_2445 pos\_2579 pos\_1159 pos\_1857 pos\_2425 pos\_2628 pos\_1966 pos\_701 pos\_2843 pos\_1616 pos\_1931 pos\_2338 pos\_1652 pos\_2682 pos\_1522 pos\_504 pos\_1517 pos\_840 pos\_461 pos\_2372 pos\_586 pos\_1565 pos\_2262 pos\_527 pos\_1601 pos\_14 pos\_2224 pos\_755 pos\_2561 pos\_695 pos\_1889 pos\_835 pos\_2695 pos\_1705 pos\_1921 pos\_1979 pos\_1307 pos\_2142 pos\_499 pos\_2154 pos\_1315 pos\_2842 pos\_1004 pos\_2664 pos\_540 pos\_1832 pos\_2085 pos\_2127 pos\_1523 pos\_2459 |
| Lipids and lipid-like molecules | Glycerolipids | 57 | pos\_1492 pos\_2480 pos\_2000 pos\_2484 pos\_2557 pos\_840 pos\_2800 pos\_1026 pos\_2578 pos\_1510 pos\_1565 pos\_1711 pos\_2382 pos\_2262 pos\_527 pos\_1147 pos\_2224 pos\_827 pos\_2679 pos\_1041 pos\_2579 pos\_2561 pos\_1159 pos\_1857 pos\_796 pos\_1426 pos\_2292 pos\_1131 pos\_2351 pos\_664 pos\_1966 pos\_800 pos\_2695 pos\_2419 pos\_1307 pos\_2560 pos\_701 pos\_1616 pos\_2154 pos\_2369 pos\_1421 pos\_1004 pos\_1790 pos\_1493 pos\_1201 pos\_1931 pos\_1998 pos\_2664 pos\_1861 pos\_1366 pos\_2356 pos\_1832 pos\_1442 pos\_1522 pos\_2127 pos\_37 pos\_2459 |
| Lipids and lipid-like molecules | Glycerophospholipids | 212 | pos\_1492 pos\_1339 pos\_1891 pos\_1965 pos\_1593 pos\_1252 pos\_1510 pos\_2382 pos\_1711 pos\_1684 pos\_1005 pos\_2195 pos\_827 pos\_560 pos\_250 pos\_627 pos\_2769 pos\_2458 pos\_2292 pos\_1247 pos\_1131 pos\_664 pos\_2277 pos\_2229 pos\_2615 pos\_2743 pos\_1470 pos\_705 pos\_2744 pos\_800 pos\_1498 pos\_2703 pos\_1865 pos\_2560 pos\_1926 pos\_1018 pos\_1125 pos\_1998 pos\_1493 pos\_1201 pos\_1570 pos\_2760 pos\_2219 pos\_2613 pos\_577 pos\_2356 pos\_1717 pos\_2632 pos\_2641 pos\_1007 pos\_1442 pos\_763 pos\_2759 pos\_37 pos\_2288 pos\_2480 pos\_2484 pos\_809 pos\_779 pos\_2800 pos\_2801 pos\_2578 pos\_2169 pos\_2485 pos\_2067 pos\_2819 pos\_718 pos\_522 pos\_2794 pos\_1143 pos\_889 pos\_2679 pos\_1041 pos\_1661 pos\_978 pos\_1271 pos\_1806 pos\_1963 pos\_796 pos\_1426 pos\_1180 pos\_2514 pos\_2351 pos\_1630 pos\_1270 pos\_2844 pos\_2725 pos\_1904 pos\_2206 pos\_604 pos\_2419 pos\_2732 pos\_2369 pos\_2559 pos\_1421 pos\_1790 pos\_1819 pos\_2263 pos\_722 pos\_1861 pos\_633 pos\_1366 pos\_932 pos\_1737 pos\_2166 pos\_1993 pos\_1604 pos\_1619 pos\_1067 pos\_554 pos\_1664 pos\_2507 pos\_2639 pos\_1207 pos\_2000 pos\_2557 pos\_1026 pos\_2236 pos\_52 pos\_1097 pos\_2068 pos\_1147 pos\_2445 pos\_2432 pos\_1833 pos\_2579 pos\_1313 pos\_1159 pos\_1857 pos\_2425 pos\_331 pos\_2628 pos\_2035 pos\_1966 pos\_1276 pos\_701 pos\_1430 pos\_1668 pos\_2843 pos\_1616 pos\_111 pos\_1931 pos\_2338 pos\_1652 pos\_2682 pos\_1522 pos\_2108 pos\_504 pos\_2683 pos\_1517 pos\_549 pos\_1115 pos\_1795 pos\_840 pos\_1716 pos\_2655 pos\_461 pos\_2372 pos\_1017 pos\_586 pos\_1876 pos\_423 pos\_1565 pos\_2341 pos\_2159 pos\_1949 pos\_2262 pos\_527 pos\_1601 pos\_14 pos\_2224 pos\_1301 pos\_1934 pos\_2151 pos\_1503 pos\_755 pos\_1145 pos\_2138 pos\_2561 pos\_695 pos\_1889 pos\_2755 pos\_1898 pos\_1257 pos\_2305 pos\_835 pos\_1161 pos\_2695 pos\_1705 pos\_1921 pos\_1979 pos\_1307 pos\_2142 pos\_499 pos\_2158 pos\_2154 pos\_1315 pos\_2842 pos\_1295 pos\_1004 pos\_2664 pos\_825 pos\_540 pos\_1832 pos\_2085 pos\_2119 pos\_1423 pos\_2127 pos\_1523 pos\_260 pos\_2459 pos\_1990 |
| Lipids and lipid-like molecules | Prenol lipids | 263 | pos\_1492 pos\_1339 pos\_1891 pos\_1965 pos\_1593 pos\_1252 pos\_1510 pos\_2382 pos\_1711 pos\_1684 pos\_1005 pos\_2195 pos\_827 pos\_560 pos\_250 pos\_627 pos\_598 pos\_1521 pos\_2769 pos\_301 pos\_2458 pos\_1445 pos\_467 pos\_2292 pos\_1247 pos\_1131 pos\_664 pos\_2277 pos\_2229 pos\_2615 pos\_2743 pos\_1470 pos\_2853 pos\_705 pos\_2744 pos\_800 pos\_1498 pos\_2703 pos\_1865 pos\_2560 pos\_1926 pos\_1018 pos\_1125 pos\_1998 pos\_1493 pos\_1201 pos\_1570 pos\_2760 pos\_2219 pos\_2613 pos\_577 pos\_2356 pos\_1717 pos\_2632 pos\_2641 pos\_1007 pos\_1543 pos\_691 pos\_1442 pos\_763 pos\_1237 pos\_2759 pos\_153 pos\_37 pos\_2288 pos\_2480 pos\_2484 pos\_809 pos\_779 pos\_2800 pos\_2801 pos\_2578 pos\_2321 pos\_446 pos\_2169 pos\_2485 pos\_2067 pos\_2819 pos\_2802 pos\_718 pos\_1046 pos\_2466 pos\_522 pos\_2794 pos\_1143 pos\_889 pos\_2679 pos\_1041 pos\_1260 pos\_1661 pos\_978 pos\_1271 pos\_1806 pos\_1963 pos\_796 pos\_1426 pos\_1180 pos\_2514 pos\_2351 pos\_1630 pos\_1270 pos\_1318 pos\_2844 pos\_2725 pos\_1799 pos\_1904 pos\_2206 pos\_604 pos\_2419 pos\_2732 pos\_2369 pos\_1282 pos\_9 pos\_2559 pos\_1421 pos\_1790 pos\_1819 pos\_2263 pos\_722 pos\_1640 pos\_1166 pos\_1861 pos\_633 pos\_1366 pos\_932 pos\_1737 pos\_2166 pos\_1993 pos\_883 pos\_1604 pos\_1210 pos\_1619 pos\_78 pos\_1067 pos\_554 pos\_1664 pos\_2507 pos\_2639 pos\_1207 pos\_2000 pos\_2557 pos\_1026 pos\_2236 pos\_52 pos\_1097 pos\_2068 pos\_2389 pos\_2825 pos\_891 pos\_769 pos\_1147 pos\_2445 pos\_2432 pos\_1833 pos\_2579 pos\_1313 pos\_1159 pos\_1857 pos\_2425 pos\_331 pos\_2628 pos\_2035 pos\_1966 pos\_199 pos\_1481 pos\_1276 pos\_701 pos\_1430 pos\_1668 pos\_2843 pos\_1616 pos\_1579 pos\_111 pos\_2588 pos\_1931 pos\_2338 pos\_220 pos\_1652 pos\_2682 pos\_1881 pos\_1522 pos\_2108 pos\_504 pos\_2683 pos\_1517 pos\_2638 pos\_549 pos\_1115 pos\_1836 pos\_1795 pos\_840 pos\_1752 pos\_1716 pos\_2655 pos\_461 pos\_2372 pos\_1017 pos\_586 pos\_1876 pos\_423 pos\_1565 pos\_2341 pos\_2010 pos\_2159 pos\_1949 pos\_2262 pos\_527 pos\_1601 pos\_14 pos\_2224 pos\_1301 pos\_1934 pos\_2151 pos\_1503 pos\_1658 pos\_755 pos\_1145 pos\_2138 pos\_2561 pos\_695 pos\_277 pos\_1889 pos\_2755 pos\_2832 pos\_687 pos\_1062 pos\_1898 pos\_1257 pos\_2305 pos\_835 pos\_1161 pos\_2729 pos\_1678 pos\_2695 pos\_1705 pos\_1921 pos\_1979 pos\_1307 pos\_2142 pos\_499 pos\_2158 pos\_2154 pos\_1315 pos\_2842 pos\_1295 pos\_155 pos\_1004 pos\_2664 pos\_825 pos\_1844 pos\_540 pos\_1251 pos\_1832 pos\_2085 pos\_2119 pos\_662 pos\_1423 pos\_2127 pos\_1523 pos\_260 pos\_720 pos\_2459 pos\_1990 |
| Lipids and lipid-like molecules | Sphingolipids | 299 | pos\_1492 pos\_1339 pos\_1891 pos\_1965 pos\_2481 pos\_1593 pos\_1252 pos\_1510 pos\_3 pos\_2382 pos\_1711 pos\_1684 pos\_1005 pos\_2195 pos\_827 pos\_560 pos\_250 pos\_627 pos\_598 pos\_1521 pos\_2769 pos\_301 pos\_2458 pos\_574 pos\_1445 pos\_1204 pos\_467 pos\_2292 pos\_1247 pos\_1131 pos\_664 pos\_2277 pos\_2229 pos\_2615 pos\_2743 pos\_1470 pos\_2853 pos\_705 pos\_2744 pos\_800 pos\_1498 pos\_2703 pos\_1865 pos\_2560 pos\_1926 pos\_1018 pos\_1125 pos\_1998 pos\_1493 pos\_1201 pos\_1570 pos\_2760 pos\_2219 pos\_2613 pos\_577 pos\_2356 pos\_1717 pos\_2632 pos\_2641 pos\_1007 pos\_1543 pos\_691 pos\_1442 pos\_763 pos\_1237 pos\_2759 pos\_1165 pos\_153 pos\_37 pos\_2288 pos\_2480 pos\_2484 pos\_809 pos\_779 pos\_2800 pos\_2801 pos\_562 pos\_2578 pos\_591 pos\_2321 pos\_446 pos\_2169 pos\_2485 pos\_1050 pos\_2067 pos\_2819 pos\_2802 pos\_718 pos\_1046 pos\_2466 pos\_522 pos\_2794 pos\_1143 pos\_889 pos\_2679 pos\_1041 pos\_1260 pos\_1661 pos\_978 pos\_1271 pos\_1806 pos\_1963 pos\_796 pos\_1426 pos\_1180 pos\_2514 pos\_2351 pos\_1630 pos\_1270 pos\_1318 pos\_2844 pos\_2725 pos\_1799 pos\_1230 pos\_57 pos\_702 pos\_1904 pos\_2206 pos\_604 pos\_2419 pos\_2732 pos\_2369 pos\_1282 pos\_9 pos\_1709 pos\_2559 pos\_1421 pos\_2268 pos\_1790 pos\_1819 pos\_2263 pos\_722 pos\_1640 pos\_1166 pos\_1861 pos\_633 pos\_1366 pos\_932 pos\_1737 pos\_2166 pos\_1993 pos\_883 pos\_1604 pos\_1210 pos\_1619 pos\_78 pos\_1067 pos\_554 pos\_1664 pos\_2507 pos\_2639 pos\_594 pos\_1751 pos\_1207 pos\_2000 pos\_2557 pos\_1248 pos\_694 pos\_1026 pos\_2236 pos\_52 pos\_1169 pos\_606 pos\_1097 pos\_2068 pos\_2389 pos\_2825 pos\_891 pos\_769 pos\_1147 pos\_2445 pos\_2432 pos\_1833 pos\_2579 pos\_1313 pos\_1159 pos\_1857 pos\_2425 pos\_331 pos\_2628 pos\_2035 pos\_1966 pos\_199 pos\_1481 pos\_1835 pos\_1276 pos\_730 pos\_701 pos\_1430 pos\_1668 pos\_2843 pos\_1616 pos\_1579 pos\_1800 pos\_111 pos\_2588 pos\_1931 pos\_2338 pos\_220 pos\_1652 pos\_2682 pos\_788 pos\_1881 pos\_1522 pos\_2108 pos\_743 pos\_504 pos\_2683 pos\_1517 pos\_2638 pos\_994 pos\_549 pos\_2308 pos\_1115 pos\_1836 pos\_1795 pos\_840 pos\_1752 pos\_1716 pos\_2655 pos\_2538 pos\_461 pos\_2372 pos\_1017 pos\_586 pos\_1876 pos\_1723 pos\_423 pos\_1565 pos\_2341 pos\_2010 pos\_1136 pos\_2159 pos\_1949 pos\_919 pos\_2262 pos\_527 pos\_1601 pos\_14 pos\_2224 pos\_1301 pos\_1934 pos\_2151 pos\_1503 pos\_1658 pos\_1100 pos\_755 pos\_1350 pos\_1145 pos\_2138 pos\_2561 pos\_695 pos\_277 pos\_1889 pos\_2755 pos\_2832 pos\_687 pos\_1062 pos\_1898 pos\_1257 pos\_2305 pos\_835 pos\_1161 pos\_2729 pos\_1678 pos\_2695 pos\_1705 pos\_1921 pos\_1979 pos\_1307 pos\_2142 pos\_499 pos\_2158 pos\_2154 pos\_1315 pos\_2842 pos\_1295 pos\_155 pos\_1004 pos\_2664 pos\_825 pos\_1844 pos\_1394 pos\_540 pos\_1251 pos\_1832 pos\_2085 pos\_2119 pos\_662 pos\_2019 pos\_1423 pos\_2127 pos\_1523 pos\_260 pos\_720 pos\_2054 pos\_2459 pos\_1990 pos\_878 |
| Lipids and lipid-like molecules | Steroids and steroid derivatives | 294 | pos\_1492 pos\_1339 pos\_1891 pos\_1965 pos\_2481 pos\_1593 pos\_1252 pos\_1510 pos\_3 pos\_2382 pos\_1711 pos\_1684 pos\_1005 pos\_2195 pos\_827 pos\_560 pos\_250 pos\_627 pos\_598 pos\_1521 pos\_2769 pos\_301 pos\_2458 pos\_574 pos\_1445 pos\_1204 pos\_467 pos\_2292 pos\_1247 pos\_1131 pos\_664 pos\_2277 pos\_2229 pos\_2615 pos\_2743 pos\_1470 pos\_2853 pos\_705 pos\_2744 pos\_800 pos\_1498 pos\_2703 pos\_1865 pos\_2560 pos\_1926 pos\_1018 pos\_1125 pos\_1998 pos\_1493 pos\_1201 pos\_1570 pos\_2760 pos\_2219 pos\_2613 pos\_577 pos\_2356 pos\_1717 pos\_2632 pos\_2641 pos\_1007 pos\_1543 pos\_691 pos\_1442 pos\_763 pos\_1237 pos\_2759 pos\_1165 pos\_153 pos\_37 pos\_2288 pos\_2480 pos\_2484 pos\_809 pos\_779 pos\_2800 pos\_2801 pos\_562 pos\_2578 pos\_591 pos\_2321 pos\_446 pos\_2169 pos\_2485 pos\_1050 pos\_2067 pos\_2819 pos\_2802 pos\_718 pos\_1046 pos\_2466 pos\_522 pos\_2794 pos\_1143 pos\_889 pos\_2679 pos\_1041 pos\_1260 pos\_1661 pos\_978 pos\_1271 pos\_1806 pos\_1963 pos\_796 pos\_1426 pos\_1180 pos\_2514 pos\_2351 pos\_1630 pos\_1270 pos\_1318 pos\_2844 pos\_2725 pos\_1799 pos\_1230 pos\_57 pos\_702 pos\_1904 pos\_2206 pos\_604 pos\_2419 pos\_2732 pos\_2369 pos\_1282 pos\_9 pos\_2559 pos\_1421 pos\_2268 pos\_1790 pos\_1819 pos\_2263 pos\_722 pos\_1640 pos\_1166 pos\_1861 pos\_633 pos\_1366 pos\_932 pos\_1737 pos\_2166 pos\_1993 pos\_883 pos\_1604 pos\_1210 pos\_1619 pos\_78 pos\_1067 pos\_554 pos\_1664 pos\_2507 pos\_2639 pos\_594 pos\_1751 pos\_1207 pos\_2000 pos\_2557 pos\_1026 pos\_2236 pos\_52 pos\_1169 pos\_606 pos\_1097 pos\_2068 pos\_2389 pos\_2825 pos\_891 pos\_769 pos\_1147 pos\_2445 pos\_2432 pos\_1833 pos\_2579 pos\_1313 pos\_1159 pos\_1857 pos\_2425 pos\_331 pos\_2628 pos\_2035 pos\_1966 pos\_199 pos\_1481 pos\_1835 pos\_1276 pos\_730 pos\_701 pos\_1430 pos\_1668 pos\_2843 pos\_1616 pos\_1579 pos\_111 pos\_2588 pos\_1931 pos\_2338 pos\_220 pos\_1652 pos\_2682 pos\_788 pos\_1881 pos\_1522 pos\_2108 pos\_743 pos\_504 pos\_2683 pos\_1517 pos\_2638 pos\_994 pos\_549 pos\_2308 pos\_1115 pos\_1836 pos\_1795 pos\_840 pos\_1752 pos\_1716 pos\_2655 pos\_461 pos\_2372 pos\_1017 pos\_586 pos\_1876 pos\_1723 pos\_423 pos\_1565 pos\_2341 pos\_2010 pos\_1136 pos\_2159 pos\_1949 pos\_919 pos\_2262 pos\_527 pos\_1601 pos\_14 pos\_2224 pos\_1301 pos\_1934 pos\_2151 pos\_1503 pos\_1658 pos\_1100 pos\_755 pos\_1350 pos\_1145 pos\_2138 pos\_2561 pos\_695 pos\_277 pos\_1889 pos\_2755 pos\_2832 pos\_687 pos\_1062 pos\_1898 pos\_1257 pos\_2305 pos\_835 pos\_1161 pos\_2729 pos\_1678 pos\_2695 pos\_1705 pos\_1921 pos\_1979 pos\_1307 pos\_2142 pos\_499 pos\_2158 pos\_2154 pos\_1315 pos\_2842 pos\_1295 pos\_155 pos\_1004 pos\_2664 pos\_825 pos\_1844 pos\_1394 pos\_540 pos\_1251 pos\_1832 pos\_2085 pos\_2119 pos\_662 pos\_2019 pos\_1423 pos\_2127 pos\_1523 pos\_260 pos\_720 pos\_2054 pos\_2459 pos\_1990 pos\_878 |
| Nucleosides, nucleotides, and analogues | 5')-dinucleotides | 8 | pos\_545 pos\_531 pos\_2182 pos\_1429 pos\_2409 pos\_2601 pos\_1025 pos\_2792 |
| Nucleosides, nucleotides, and analogues | Purine nucleosides | 1 | pos\_1025 |
| Nucleosides, nucleotides, and analogues | Purine nucleotides | 9 | pos\_545 pos\_531 pos\_2182 pos\_1429 pos\_2409 pos\_2601 pos\_1025 pos\_2792 pos\_602 |
| Nucleosides, nucleotides, and analogues | Pyrimidine nucleosides | 5 | pos\_531 pos\_2182 pos\_1429 pos\_1025 pos\_2792 |
| Nucleosides, nucleotides, and analogues | Pyrimidine nucleotides | 6 | pos\_531 pos\_2182 pos\_1429 pos\_2409 pos\_1025 pos\_2792 |
| Organic Polymers | Polypeptides | 1 | pos\_2323 |
| Organic acids and derivatives | Carboximidic acids and derivatives | 87 | pos\_782 pos\_2624 pos\_2487 pos\_807 pos\_759 pos\_770 pos\_2 pos\_733 pos\_615 pos\_2310 pos\_2118 pos\_709 pos\_671 pos\_1674 pos\_650 pos\_251 pos\_1439 pos\_767 pos\_1267 pos\_2506 pos\_1092 pos\_2004 pos\_1675 pos\_1696 pos\_674 pos\_1573 pos\_2813 pos\_2447 pos\_2816 pos\_1972 pos\_829 pos\_766 pos\_1720 pos\_1896 pos\_717 pos\_814 pos\_462 pos\_1160 pos\_1924 pos\_1518 pos\_1416 pos\_1323 pos\_535 pos\_1369 pos\_592 pos\_2658 pos\_1195 pos\_2687 pos\_1927 pos\_2716 pos\_2136 pos\_1581 pos\_1264 pos\_963 pos\_2274 pos\_536 pos\_495 pos\_1215 pos\_2233 pos\_556 pos\_2829 pos\_1085 pos\_1425 pos\_916 pos\_819 pos\_2438 pos\_569 pos\_1941 pos\_999 pos\_1329 pos\_144 pos\_2012 pos\_614 pos\_1036 pos\_1428 pos\_1027 pos\_1419 pos\_980 pos\_2599 pos\_970 pos\_1396 pos\_1448 pos\_1479 pos\_926 pos\_1913 pos\_1250 pos\_2491 |
| Organic acids and derivatives | Carboxylic acids and derivatives | 77 | pos\_2624 pos\_807 pos\_759 pos\_770 pos\_2 pos\_733 pos\_615 pos\_2310 pos\_2118 pos\_709 pos\_671 pos\_1674 pos\_650 pos\_251 pos\_1439 pos\_767 pos\_1267 pos\_2506 pos\_1092 pos\_2004 pos\_1675 pos\_1696 pos\_1573 pos\_2813 pos\_2447 pos\_2816 pos\_1972 pos\_829 pos\_766 pos\_1720 pos\_1896 pos\_814 pos\_462 pos\_1160 pos\_1924 pos\_1518 pos\_1416 pos\_1323 pos\_535 pos\_1369 pos\_592 pos\_1195 pos\_2687 pos\_1927 pos\_2716 pos\_2136 pos\_1581 pos\_1264 pos\_2274 pos\_536 pos\_495 pos\_1215 pos\_2233 pos\_556 pos\_2829 pos\_1085 pos\_916 pos\_819 pos\_2438 pos\_569 pos\_1941 pos\_999 pos\_1329 pos\_144 pos\_2012 pos\_614 pos\_1036 pos\_1428 pos\_1027 pos\_1419 pos\_2599 pos\_1396 pos\_1448 pos\_1479 pos\_926 pos\_1913 pos\_1250 |
| Organic acids and derivatives | Hydroxy acids and derivatives | 85 | pos\_782 pos\_2624 pos\_2487 pos\_807 pos\_759 pos\_770 pos\_2 pos\_733 pos\_615 pos\_2310 pos\_2118 pos\_709 pos\_671 pos\_1674 pos\_650 pos\_251 pos\_1439 pos\_767 pos\_1267 pos\_2506 pos\_1092 pos\_2004 pos\_1675 pos\_1696 pos\_674 pos\_1573 pos\_2813 pos\_2447 pos\_2816 pos\_1972 pos\_829 pos\_766 pos\_1720 pos\_1896 pos\_717 pos\_814 pos\_462 pos\_1160 pos\_1924 pos\_1518 pos\_1416 pos\_1323 pos\_535 pos\_1369 pos\_592 pos\_1195 pos\_2687 pos\_1927 pos\_2716 pos\_2136 pos\_1581 pos\_1264 pos\_963 pos\_2274 pos\_536 pos\_495 pos\_1215 pos\_2233 pos\_556 pos\_2829 pos\_1085 pos\_1425 pos\_916 pos\_819 pos\_2438 pos\_569 pos\_1941 pos\_999 pos\_1329 pos\_144 pos\_2012 pos\_614 pos\_1036 pos\_1428 pos\_1027 pos\_1419 pos\_2599 pos\_970 pos\_1396 pos\_1448 pos\_1479 pos\_926 pos\_1913 pos\_1250 pos\_2491 |
| Organic acids and derivatives | Keto acids and derivatives | 3 | pos\_1027 pos\_1195 pos\_2687 |
| Organic acids and derivatives | Organic phosphoric acids and derivatives | 86 | pos\_782 pos\_2624 pos\_2487 pos\_807 pos\_759 pos\_770 pos\_2 pos\_733 pos\_615 pos\_2310 pos\_2118 pos\_709 pos\_671 pos\_1674 pos\_650 pos\_251 pos\_1439 pos\_767 pos\_1267 pos\_2506 pos\_1092 pos\_2004 pos\_1675 pos\_1696 pos\_674 pos\_1573 pos\_2813 pos\_2447 pos\_2816 pos\_1972 pos\_829 pos\_766 pos\_1720 pos\_1896 pos\_717 pos\_814 pos\_462 pos\_1160 pos\_1924 pos\_1518 pos\_1416 pos\_1323 pos\_535 pos\_1369 pos\_592 pos\_2658 pos\_1195 pos\_2687 pos\_1927 pos\_2716 pos\_2136 pos\_1581 pos\_1264 pos\_963 pos\_2274 pos\_536 pos\_495 pos\_1215 pos\_2233 pos\_556 pos\_2829 pos\_1085 pos\_1425 pos\_916 pos\_819 pos\_2438 pos\_569 pos\_1941 pos\_999 pos\_1329 pos\_144 pos\_2012 pos\_614 pos\_1036 pos\_1428 pos\_1027 pos\_1419 pos\_2599 pos\_970 pos\_1396 pos\_1448 pos\_1479 pos\_926 pos\_1913 pos\_1250 pos\_2491 |
| Organic acids and derivatives | Organic sulfuric acids and derivatives | 82 | pos\_782 pos\_2624 pos\_2487 pos\_807 pos\_759 pos\_770 pos\_2 pos\_733 pos\_615 pos\_2310 pos\_2118 pos\_709 pos\_671 pos\_1674 pos\_650 pos\_251 pos\_1439 pos\_767 pos\_1267 pos\_2506 pos\_1092 pos\_2004 pos\_1675 pos\_1696 pos\_1573 pos\_2813 pos\_2447 pos\_2816 pos\_1972 pos\_829 pos\_766 pos\_1720 pos\_1896 pos\_814 pos\_462 pos\_1160 pos\_1924 pos\_1518 pos\_1416 pos\_1323 pos\_535 pos\_1369 pos\_592 pos\_1195 pos\_2687 pos\_1927 pos\_2716 pos\_2136 pos\_1581 pos\_1264 pos\_963 pos\_2274 pos\_536 pos\_495 pos\_1215 pos\_2233 pos\_556 pos\_2829 pos\_1085 pos\_1425 pos\_916 pos\_819 pos\_2438 pos\_569 pos\_1941 pos\_999 pos\_1329 pos\_144 pos\_2012 pos\_614 pos\_1036 pos\_1428 pos\_1027 pos\_1419 pos\_2599 pos\_970 pos\_1396 pos\_1448 pos\_1479 pos\_926 pos\_1913 pos\_1250 |
| Organic acids and derivatives | Peptidomimetics | 80 | pos\_2624 pos\_807 pos\_759 pos\_770 pos\_2 pos\_733 pos\_615 pos\_2310 pos\_2118 pos\_709 pos\_671 pos\_1674 pos\_650 pos\_251 pos\_1439 pos\_767 pos\_1267 pos\_2506 pos\_1092 pos\_2004 pos\_1675 pos\_1696 pos\_1573 pos\_2813 pos\_2447 pos\_2816 pos\_1972 pos\_829 pos\_766 pos\_1720 pos\_1896 pos\_814 pos\_462 pos\_1160 pos\_1924 pos\_1518 pos\_1416 pos\_1323 pos\_535 pos\_1369 pos\_592 pos\_1195 pos\_2687 pos\_1927 pos\_2716 pos\_2136 pos\_1581 pos\_1264 pos\_963 pos\_2274 pos\_536 pos\_495 pos\_1215 pos\_2233 pos\_556 pos\_2829 pos\_1085 pos\_1425 pos\_916 pos\_819 pos\_2438 pos\_569 pos\_1941 pos\_999 pos\_1329 pos\_144 pos\_2012 pos\_614 pos\_1036 pos\_1428 pos\_1027 pos\_1419 pos\_2599 pos\_970 pos\_1396 pos\_1448 pos\_1479 pos\_926 pos\_1913 pos\_1250 |
| Organic nitrogen compounds | Organonitrogen compounds | 16 | pos\_1699 pos\_1713 pos\_514 pos\_1438 pos\_1662 pos\_2541 pos\_813 pos\_884 pos\_1001 pos\_866 pos\_1275 pos\_2730 pos\_1 pos\_879 pos\_300 pos\_2204 |
| Organic oxygen compounds | Organooxygen compounds | 38 | pos\_816 pos\_543 pos\_1241 pos\_1444 pos\_976 pos\_593 pos\_1431 pos\_2258 pos\_2087 pos\_21 pos\_1608 pos\_638 pos\_2116 pos\_542 pos\_799 pos\_1440 pos\_2785 pos\_1285 pos\_752 pos\_2731 pos\_2379 pos\_2656 pos\_863 pos\_1009 pos\_472 pos\_2531 pos\_1902 pos\_1129 pos\_477 pos\_2013 pos\_634 pos\_2649 pos\_2590 pos\_77 pos\_2363 pos\_1842 pos\_1525 pos\_19 |
| Organohalogen compounds | Alkyl halides | 1 | pos\_154 |
| Organoheterocyclic compounds | Azaphilones | 20 | pos\_1729 pos\_2720 pos\_1286 pos\_1986 pos\_1153 pos\_1588 pos\_2162 pos\_172 pos\_597 pos\_764 pos\_431 pos\_2096 pos\_1541 pos\_564 pos\_152 pos\_1740 pos\_2028 pos\_2218 pos\_118 pos\_1569 |
| Organoheterocyclic compounds | Azepines | 29 | pos\_1729 pos\_2720 pos\_2322 pos\_1286 pos\_1986 pos\_1153 pos\_1588 pos\_2545 pos\_298 pos\_2162 pos\_172 pos\_597 pos\_764 pos\_2205 pos\_2539 pos\_431 pos\_2096 pos\_2316 pos\_2635 pos\_1541 pos\_564 pos\_152 pos\_1740 pos\_625 pos\_2028 pos\_2218 pos\_2364 pos\_118 pos\_1569 |
| Organoheterocyclic compounds | Azoles | 32 | pos\_1729 pos\_2720 pos\_2322 pos\_1286 pos\_1986 pos\_1153 pos\_1588 pos\_2545 pos\_298 pos\_2162 pos\_172 pos\_597 pos\_764 pos\_481 pos\_2205 pos\_2539 pos\_431 pos\_2096 pos\_2316 pos\_2635 pos\_1541 pos\_564 pos\_152 pos\_1740 pos\_625 pos\_2028 pos\_590 pos\_2218 pos\_2364 pos\_118 pos\_1569 pos\_61 |
| Organoheterocyclic compounds | Benzimidazoles | 36 | pos\_1729 pos\_2720 pos\_2322 pos\_1286 pos\_1986 pos\_1153 pos\_1588 pos\_2545 pos\_298 pos\_2162 pos\_172 pos\_929 pos\_668 pos\_597 pos\_683 pos\_764 pos\_676 pos\_481 pos\_2205 pos\_2539 pos\_431 pos\_2096 pos\_2316 pos\_2635 pos\_1541 pos\_564 pos\_152 pos\_1740 pos\_625 pos\_2028 pos\_590 pos\_2218 pos\_2364 pos\_118 pos\_1569 pos\_61 |
| Organoheterocyclic compounds | Benzodioxanes | 13 | pos\_2720 pos\_597 pos\_2028 pos\_1286 pos\_152 pos\_2218 pos\_1153 pos\_1541 pos\_118 pos\_1569 pos\_1588 pos\_564 pos\_764 |
| Organoheterocyclic compounds | Benzodioxoles | 38 | pos\_1729 pos\_2720 pos\_2322 pos\_1286 pos\_69 pos\_1986 pos\_1153 pos\_761 pos\_1588 pos\_2545 pos\_298 pos\_2162 pos\_172 pos\_929 pos\_668 pos\_597 pos\_683 pos\_764 pos\_676 pos\_481 pos\_2205 pos\_2539 pos\_431 pos\_2096 pos\_2316 pos\_2635 pos\_1541 pos\_564 pos\_152 pos\_1740 pos\_625 pos\_2028 pos\_590 pos\_2218 pos\_2364 pos\_118 pos\_1569 pos\_61 |
| Organoheterocyclic compounds | Benzopyrans | 28 | pos\_1729 pos\_2720 pos\_2322 pos\_1286 pos\_1986 pos\_1153 pos\_1588 pos\_2545 pos\_298 pos\_2162 pos\_172 pos\_597 pos\_764 pos\_2205 pos\_2539 pos\_431 pos\_2096 pos\_2316 pos\_1541 pos\_564 pos\_152 pos\_1740 pos\_625 pos\_2028 pos\_2218 pos\_2364 pos\_118 pos\_1569 |
| Organoheterocyclic compounds | Diazinanes | 16 | pos\_1729 pos\_2720 pos\_597 pos\_2028 pos\_1286 pos\_152 pos\_431 pos\_2218 pos\_1153 pos\_1541 pos\_118 pos\_1569 pos\_1588 pos\_564 pos\_764 pos\_172 |
| Organoheterocyclic compounds | Diazines | 18 | pos\_1729 pos\_2720 pos\_2162 pos\_597 pos\_2028 pos\_1286 pos\_152 pos\_431 pos\_2218 pos\_1153 pos\_1541 pos\_118 pos\_1569 pos\_1588 pos\_564 pos\_764 pos\_1740 pos\_172 |
| Organoheterocyclic compounds | Furans | 25 | pos\_1729 pos\_2720 pos\_2322 pos\_1286 pos\_1986 pos\_1153 pos\_1588 pos\_2545 pos\_2162 pos\_172 pos\_597 pos\_764 pos\_2205 pos\_431 pos\_2096 pos\_2316 pos\_1541 pos\_564 pos\_152 pos\_1740 pos\_625 pos\_2028 pos\_2218 pos\_118 pos\_1569 |
| Organoheterocyclic compounds | Furofurans | 31 | pos\_1729 pos\_2720 pos\_2322 pos\_1286 pos\_1986 pos\_1153 pos\_1588 pos\_2545 pos\_298 pos\_2162 pos\_172 pos\_597 pos\_764 pos\_481 pos\_2205 pos\_2539 pos\_431 pos\_2096 pos\_2316 pos\_2635 pos\_1541 pos\_564 pos\_152 pos\_1740 pos\_625 pos\_2028 pos\_590 pos\_2218 pos\_2364 pos\_118 pos\_1569 |
| Organoheterocyclic compounds | Imidazopyrimidines | 12 | pos\_2720 pos\_597 pos\_2028 pos\_1286 pos\_152 pos\_2218 pos\_1153 pos\_1541 pos\_118 pos\_1569 pos\_564 pos\_764 |
| Organoheterocyclic compounds | Indoles and derivatives | 4 | pos\_1153 pos\_1541 pos\_764 pos\_118 |
| Organoheterocyclic compounds | Lactones | 37 | pos\_1729 pos\_2720 pos\_2322 pos\_1286 pos\_69 pos\_1986 pos\_1153 pos\_1588 pos\_2545 pos\_298 pos\_2162 pos\_172 pos\_929 pos\_668 pos\_597 pos\_683 pos\_764 pos\_676 pos\_481 pos\_2205 pos\_2539 pos\_431 pos\_2096 pos\_2316 pos\_2635 pos\_1541 pos\_564 pos\_152 pos\_1740 pos\_625 pos\_2028 pos\_590 pos\_2218 pos\_2364 pos\_118 pos\_1569 pos\_61 |
| Organoheterocyclic compounds | Naphthopyrans | 24 | pos\_1729 pos\_2720 pos\_1286 pos\_1986 pos\_1153 pos\_1588 pos\_2545 pos\_2162 pos\_172 pos\_597 pos\_764 pos\_2205 pos\_431 pos\_2096 pos\_2316 pos\_1541 pos\_564 pos\_152 pos\_1740 pos\_625 pos\_2028 pos\_2218 pos\_118 pos\_1569 |
| Organoheterocyclic compounds | Piperidines | 34 | pos\_1729 pos\_2720 pos\_2322 pos\_1286 pos\_1986 pos\_1153 pos\_1588 pos\_2545 pos\_298 pos\_2162 pos\_172 pos\_929 pos\_668 pos\_597 pos\_764 pos\_481 pos\_2205 pos\_2539 pos\_431 pos\_2096 pos\_2316 pos\_2635 pos\_1541 pos\_564 pos\_152 pos\_1740 pos\_625 pos\_2028 pos\_590 pos\_2218 pos\_2364 pos\_118 pos\_1569 pos\_61 |
| Organoheterocyclic compounds | Pteridines and derivatives | 19 | pos\_1729 pos\_2720 pos\_1286 pos\_1986 pos\_1153 pos\_1588 pos\_2162 pos\_172 pos\_597 pos\_764 pos\_431 pos\_1541 pos\_564 pos\_152 pos\_1740 pos\_2028 pos\_2218 pos\_118 pos\_1569 |
| Organoheterocyclic compounds | Pyrans | 26 | pos\_1729 pos\_2720 pos\_2322 pos\_1286 pos\_1986 pos\_1153 pos\_1588 pos\_2545 pos\_2162 pos\_172 pos\_597 pos\_764 pos\_2205 pos\_431 pos\_2096 pos\_2316 pos\_1541 pos\_564 pos\_152 pos\_1740 pos\_625 pos\_2028 pos\_2218 pos\_2364 pos\_118 pos\_1569 |
| Organoheterocyclic compounds | Pyridines and derivatives | 22 | pos\_1729 pos\_2720 pos\_1286 pos\_1986 pos\_1153 pos\_1588 pos\_2545 pos\_2162 pos\_172 pos\_597 pos\_764 pos\_2205 pos\_431 pos\_2096 pos\_1541 pos\_564 pos\_152 pos\_1740 pos\_2028 pos\_2218 pos\_118 pos\_1569 |
| Organoheterocyclic compounds | Pyrrolidines | 14 | pos\_2720 pos\_597 pos\_2028 pos\_1286 pos\_152 pos\_2218 pos\_1153 pos\_1541 pos\_118 pos\_1569 pos\_1588 pos\_564 pos\_764 pos\_172 |
| Organoheterocyclic compounds | Quinolines and derivatives | 35 | pos\_1729 pos\_2720 pos\_2322 pos\_1286 pos\_1986 pos\_1153 pos\_1588 pos\_2545 pos\_298 pos\_2162 pos\_172 pos\_929 pos\_668 pos\_597 pos\_764 pos\_676 pos\_481 pos\_2205 pos\_2539 pos\_431 pos\_2096 pos\_2316 pos\_2635 pos\_1541 pos\_564 pos\_152 pos\_1740 pos\_625 pos\_2028 pos\_590 pos\_2218 pos\_2364 pos\_118 pos\_1569 pos\_61 |
| Organoheterocyclic compounds | Tetrahydrofurans | 30 | pos\_1729 pos\_2720 pos\_2322 pos\_1286 pos\_1986 pos\_1153 pos\_1588 pos\_2545 pos\_298 pos\_2162 pos\_172 pos\_597 pos\_764 pos\_481 pos\_2205 pos\_2539 pos\_431 pos\_2096 pos\_2316 pos\_2635 pos\_1541 pos\_564 pos\_152 pos\_1740 pos\_625 pos\_2028 pos\_2218 pos\_2364 pos\_118 pos\_1569 |
| Organoheterocyclic compounds | Tetrahydroisoquinolines | 1 | pos\_1153 |
| Organoheterocyclic compounds | Tetrapyrroles and derivatives | 8 | pos\_2720 pos\_1286 pos\_152 pos\_2218 pos\_1153 pos\_1541 pos\_118 pos\_764 |
| Organosulfur compounds | Thioethers | 2 | pos\_537 pos\_325 |
| Phenylpropanoids and polyketides | 3,4-dihydrocoumarins | 17 | pos\_149 pos\_936 pos\_387 pos\_16 pos\_2670 pos\_2279 pos\_1193 pos\_2493 pos\_1687 pos\_1763 pos\_1831 pos\_1289 pos\_1500 pos\_203 pos\_276 pos\_173 pos\_117 |
| Phenylpropanoids and polyketides | Alpha-methyldeoxybenzoin flavonoids | 20 | pos\_149 pos\_1193 pos\_203 pos\_1289 pos\_656 pos\_936 pos\_16 pos\_173 pos\_2670 pos\_1763 pos\_1500 pos\_2493 pos\_387 pos\_648 pos\_2830 pos\_1687 pos\_117 pos\_2279 pos\_276 pos\_1831 |
| Phenylpropanoids and polyketides | Cinnamaldehydes | 19 | pos\_149 pos\_1193 pos\_203 pos\_1289 pos\_936 pos\_16 pos\_173 pos\_2670 pos\_1763 pos\_1500 pos\_2493 pos\_387 pos\_648 pos\_2830 pos\_1687 pos\_117 pos\_2279 pos\_276 pos\_1831 |
| Phenylpropanoids and polyketides | Cinnamic acids and derivatives | 9 | pos\_149 pos\_387 pos\_16 pos\_1193 pos\_2493 pos\_1687 pos\_1289 pos\_1500 pos\_117 |
| Phenylpropanoids and polyketides | Coumarins and derivatives | 7 | pos\_149 pos\_387 pos\_1193 pos\_2493 pos\_1289 pos\_1500 pos\_117 |
| Phenylpropanoids and polyketides | Flavonoids | 14 | pos\_149 pos\_936 pos\_387 pos\_16 pos\_1193 pos\_2493 pos\_1687 pos\_1763 pos\_1831 pos\_1289 pos\_1500 pos\_203 pos\_173 pos\_117 |
| Phenylpropanoids and polyketides | Isoflavonoids | 3 | pos\_1289 pos\_1193 pos\_117 |
| Phenylpropanoids and polyketides | Linear 1,3-diarylpropanoids | 1 | pos\_117 |
| Phenylpropanoids and polyketides | Macrolactams | 16 | pos\_149 pos\_936 pos\_387 pos\_16 pos\_2670 pos\_2279 pos\_1193 pos\_2493 pos\_1687 pos\_1763 pos\_1831 pos\_1289 pos\_1500 pos\_203 pos\_173 pos\_117 |
| Phenylpropanoids and polyketides | Macrolides and analogues | 18 | pos\_149 pos\_936 pos\_387 pos\_16 pos\_2670 pos\_2279 pos\_1193 pos\_2493 pos\_1687 pos\_1763 pos\_1831 pos\_1289 pos\_1500 pos\_203 pos\_276 pos\_2830 pos\_173 pos\_117 |
| Phenylpropanoids and polyketides | Phenylpropanoic acids | 15 | pos\_149 pos\_936 pos\_387 pos\_16 pos\_2279 pos\_1193 pos\_2493 pos\_1687 pos\_1763 pos\_1831 pos\_1289 pos\_1500 pos\_203 pos\_173 pos\_117 |
| Phenylpropanoids and polyketides | Stilbenes | 4 | pos\_1289 pos\_1193 pos\_149 pos\_117 |
| Phenylpropanoids and polyketides | oids | 10 | pos\_149 pos\_387 pos\_16 pos\_1193 pos\_2493 pos\_1687 pos\_1289 pos\_1500 pos\_173 pos\_117 |
